# SEPT9_v2, frequently silenced by promoter hypermethylation, exerts anti-tumor functions through inactivation of Wnt/β-catenin signaling pathway via miR92b-3p/FZD10 in nasopharyngeal carcinoma cells

**DOI:** 10.1186/s13148-020-00833-5

**Published:** 2020-03-05

**Authors:** Yu Jiang, Lei Liu, Qin Xiang, Xiaoqian He, Yan Wang, Dishu Zhou, Can Zou, Qian Chen, Mingyu Peng, Jin He, Xianyao Jiang, Tingxiu Xiang, Yucheng Yang

**Affiliations:** 1 Department of Otorhinolaryngology, The First Affiliated Hospital of Chongqing Medical University, No. 1 Youyi Road, Yuzhong District, Chongqing, 400016 China; 2 Key Laboratory of Molecular Oncology and Epigenetics, The First Affiliated Hospital of Chongqing Medical University, No. 1 Youyi Road, Yuzhong District, Chongqing, 400016 China

**Keywords:** SEPT9_v2, nasopharyngeal carcinoma, Wnt/β-catenin, FZD10, miR92b-3p

## Abstract

**Background:**

Nasopharyngeal carcinoma tends to present at an advanced stage because the primary anatomic site is located in a less visible area and its clinical symptoms are nonspecific. Prognosis of advanced nasopharyngeal carcinoma cases remains disappointing. SEPT9 is a methylation-based biomarker approved by the US Food and Drug Administration for colorectal cancer screening and diagnosis. Interestingly, downregulation of SEPT9, especially SEPT9_v2, mediated by promoter hypermethylation has been also detected in head and neck squamous cell carcinoma than in head and neck squamous epithelium, while other SEPT9 variants did not. These reasons above indicate a crucial role of SEPT9_v2 in cancer progression. Therefore, we address the methylation status of SEPT9_v2 in nasopharyngeal carcinoma and explore the role of SEPT9_v2 in nasopharyngeal carcinoma proliferation and cancer progression.

**Results:**

SEPT9_v2 expression was found to be downregulated via promoter methylation in nasopharyngeal carcinoma cell lines and tissues. Ectopic expression of SEPT9_v2 induced G0/G1 cell cycle arrest and apoptosis, which exerted an inhibitory effect in cell proliferation and colony formation. Additionally, nasopharyngeal carcinoma cell migration and invasion were shown to be inhibited by SEPT9_v2. Furthermore, our data suggested that SEPT9_v2 inhibits proliferation and migration of nasopharyngeal carcinoma cells through inactivation of the Wnt/β-catenin signaling pathway via miR92b-3p/FZD10.

**Conclusions:**

This study delineates SEPT9_v2, frequently silenced by promoter hypermethylation, exerts anti-tumor functions through inactivation of the Wnt/β-catenin signaling pathway via miR92b-3p/FZD10 in nasopharyngeal carcinoma cells and, hence, SEPT9_v2 may be a promising therapeutic target and biomarker for nasopharyngeal carcinoma.

## Background

A reliable cancer biomarker can be used for diagnosis, prognosis, prediction of treatment efficacy and toxicity, and recurrence. Thus, a great deal of effort has been devoted to the exploration of new cancer biomarkers [1]. Epigenetic alterations are already being incorporated as valuable candidates in the biomarker field. Furthermore, their reversible nature offers a promising opportunity to ameliorate disease symptoms by using epigenetic-based therapy. Some biomarkers have been approved by the US Food and Drug Administration (FDA) for cancer diagnosis, prognosis, or response to therapy [2]. Among these FDA-approved methylation-based biomarkers, SEPT9 is of particular note. In 2011, Toth et al. reported first the influence of methylated SEPT9 gene on RNA and protein levels in colorectal cancer [3]. Afterward, the SEPT9-methylated DNA test was identified to have an overall sensitivity for colorectal cancer of 90% and specificity of 88% [4]. A further study which detected the methylated SEPT9 in the tissue and plasma of colorectal patients revealed that tissue levels of methylated SEPT9 alone are not sufficient to predict methylated SEPT9 levels in the plasma. Additional parameters including the amount of cfDNA in the plasma appear to also play a role [5]. After that, a blood-based testing kit named Epi proColon® 2.0 CE was designed to aid in the early detection of colorectal cancer [6]. It was the first blood-based test approved by the FDA as a colorectal screening test [7]. SEPT9 is a member of the septin family, which is an evolutionarily conserved family of GTPases. Septins are associated with a number of cellular processes including cell division and are linked to various diseases, especially cancer [8]. For example, Koch et al. found that post-transcriptional Wnt signaling regulates the sperm diffusion barrier function via septin4 [9]. SEPT9, located at chromosome 17q25.3, was the first septin implicated in cancer and is a fusion partner gene of MLL [10]. It can produce up to 18 mRNA variants encoding 15 polypeptides. A number of compelling evidences have shown specific SEPT9 variants have different effects on oncogenesis. SEPT9_v1 has been reported to be an oncogene for breast cancer [11], head and neck squamous cell carcinoma (HNSC) [12], prostate cancer [13], and ovarian cancer [14]. Downregulation of SEPT9_v3 was detected in breast cancer patients [15].

Interestingly, downregulation of SEPT9 mediated by promoter hypermethylation has been detected not only in colorectal cancer but also in breast cancer [16] and head and neck squamous cell carcinoma [17]. More intriguingly, aberrant SEPT9 DNA methylation in colorectal cancer was found to be restricted to a single CpG island [18]. Then, we explored the SEPT9 methylation and gene expression using the MethHC database. We found that only SEPT9_v2 had significantly higher methylation levels in HNSC, while other SEPT9 variants did not. Wasserkort et al. had revealed that disease-associated aberrant methylation was seen exclusively in an intragenic CpG island (CGI3), while the other regions in SEPT9 did not show detectable alterations in their methylation status. Meanwhile, both the genomic location of CGI3 and the presence of a transcription start site in this specific region strongly suggest a direct role in the regulation of SEPT9_v2 [18]. These reasons above indicate a crucial role of SEPT9_v2 in cancer progression. However, despite published researches that have revealed the diagnostic value of methylated SEPT9 in colorectal cancer, a detailed analysis that explores the function and mechanism of SEPT9_v2 in cancer progression is lacking. Nasopharyngeal carcinoma (NPC), a head and neck cancer with high incidence in China and Southeast Asia, is characterized with a high invasive and metastatic nature [19]. NPC tends to present at an advanced stage because the primary anatomic site is located in a less visible area and its clinical symptoms are nonspecific [20]. Despite the advances of radiotherapy and chemotherapy, survival has not been evidently improved in advanced and poorly differentiated NPC [21]. Therefore, it is important to understand the molecular basis for NPC pathogenesis and to identify novel biomarkers for diagnosis and treatment of NPC. The etiology of NPC is not fully understood, but it is proven to be associated with Epstein-Barr virus infection, genome, and environment. A variety of epigenetic modifications also influence NPC substantially. Among these modifications, DNA methylation has a significant impact on the expression of genes involved in NPC development and progression. Recently, hypermethylation of tumor suppressor genes, including p16, RASSF1, CDKN2A, CDH1, and miR-148a, has been detected in NPC, which can impact a variety of pathways including Wnt/β-catenin and MAPK/ERK [22].

We hypothesized that SEPT9_v2 is a tumor suppressor, and the promoter of this gene may be frequently hypermethylated in cancers including NPC. Hence, in the present study, we first investigated the methylation status of SEPT9_v2 in NPC and identified its role in NPC progression.

## Results

### SEPT9_v2 expression is downregulated in NPC tissues and cell lines by promoter methylation

**Fig. 1:**
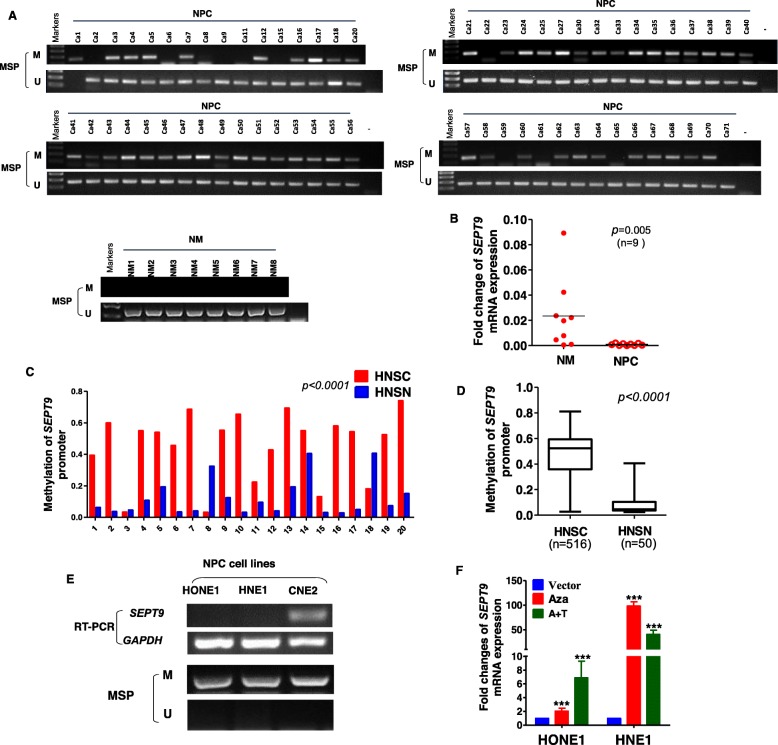
The expression levels and promoter methylation levels of SEPT9_v2 in NPC tissues, HNSC tissues, and cell lines. NM tissues and HNSN tissues were used as controls. **a** The promoter methylation level of SEPT9_v2 in 71 NPC tissues was significantly higher in comparison with 8 normal nasal mucosal tissues by MSP. **b** SEPT9_v2 expression in 9 human nasopharyngeal carcinomas and 9 NM tissues detected by qPCR. **c** SEPT9_v2 promoter methylation in 20 paired HNSC and HNSN tissue samples from the MethHC database. **d** SEPT9_v2 promoter methylation in 516 HNSC samples and 50 HNSN samples. **e** SEPT9_v2 mRNA expression and methylation status in HONE1 and HNE1 cell lines were detected by RT-PCR and MSP analysis. SEPT9_v2 was downregulated and hypermethylated in HONE1 and HNE1 cell lines. GAPDH was used as an input control. GAPDH was used as an input control. **f** qPCR detected SEPT9_v2 mRNA expression in HONE1 and HNE1 cell lines treated with Aza (A) without or with TSA (T). Error bars mean standard deviation (SD); values are presented as the mean ± SD of at least three independent experiments. Aza, 5-aza-2′-deoxycytidine; HNSC, head and neck squamous cell carcinomas; HNSN, head and neck squamous epithelium; MSP, methylation-specific polymerase chain reaction; M, methylated; NPC, nasopharyngeal carcinoma; NM, normal nasal mucosal; SD, standard deviation; U, unmethylated; RT-PCR, reverse transcription polymerase chain reaction. *p < 0.05, **p < 0.01, ***p < 0.001

To investigate the role of SEPT9_v2 in NPC, we first set out to examine the promoter methylation level of SEPT9_v2 in 71 NPC and 8 normal nasal mucosal (NM) tissues by methylation-specific PCR (MSP). A higher promoter methylation level of SEPT9_v2 was observed in NPC than in NM tissues (Fig. 1a). By statistical analysis, hypermethylation was detected in 82% (58/71) nasopharyngeal carcinoma tissue samples while in 0 (0/8) normal nasal mucosal tissue samples (Table 1). Then, we performed quantitative reverse transcription polymerase chain reaction (qPCR) to verify the expression levels of SEPT9_v2 in human NPC tissues and in NM tissues. SEPT9_v2 was less expressed in nasopharyngeal carcinoma tissues (*n* = 9) than in NM tissues (*n* = 9) (Fig. 1b). Importantly, twenty tissue pairs from the MethHC dataset [23] also showed high promoter methylation levels in HNSC tissues (Fig. 1c). By the use of the MethHC database, we found that SEPT9_v2 had significantly higher methylation levels in HNSC (*n* = 516) than in head and neck squamous epithelium (HNSN) (*n* = 50) (Fig. 1d), while other SEPT9 variants did not (Additional file 1: Figure S1A–F). The results confirmed a crucial role of SEPT9_v2 in HNSC. In NPC cell lines, a similar trend was identified by reverse transcription polymerase chain reaction (RT-PCR) and methylation-specific PCR (MSP) (Fig. 1e). To further verify whether promoter methylation contributed to the downregulation of SEPT9_v2 expression levels, treatment of cells with 5-Aza-2′-deoxycytidine (Aza) with or without trichostatin A (TSA) was conducted and mRNA levels of SEPT9_v2 were strongly increased after treatment, as compared to untreated cells, indicating that SEPT9_v2 expression was downregulated by promoter methylation in these cell lines (Fig. 1f). These results were consistent with that SEPT9_v2 expression was downregulated via the promoter methylation in nasopharyngeal carcinoma.

**Table 1:** Methylation status of the SEPT9_v2 promoter in NPC and normal nasal mucosal tissues (NM)

Samples	SEPT9_v2 promoter		Frequency of methylation	*p* value (*χ*^2^ test)
	methylated	unmethylated		
NPC (*n* = 71)	58	13	82%	*p* < 0.001
NM (*n* = 8)	0	8	0	

### Ectopic expression of SEPT9_v2 inhibits cell proliferation by inducing G0/G1 cell cycle arrest and apoptosis in NPC cell lines

**Fig. 2:**
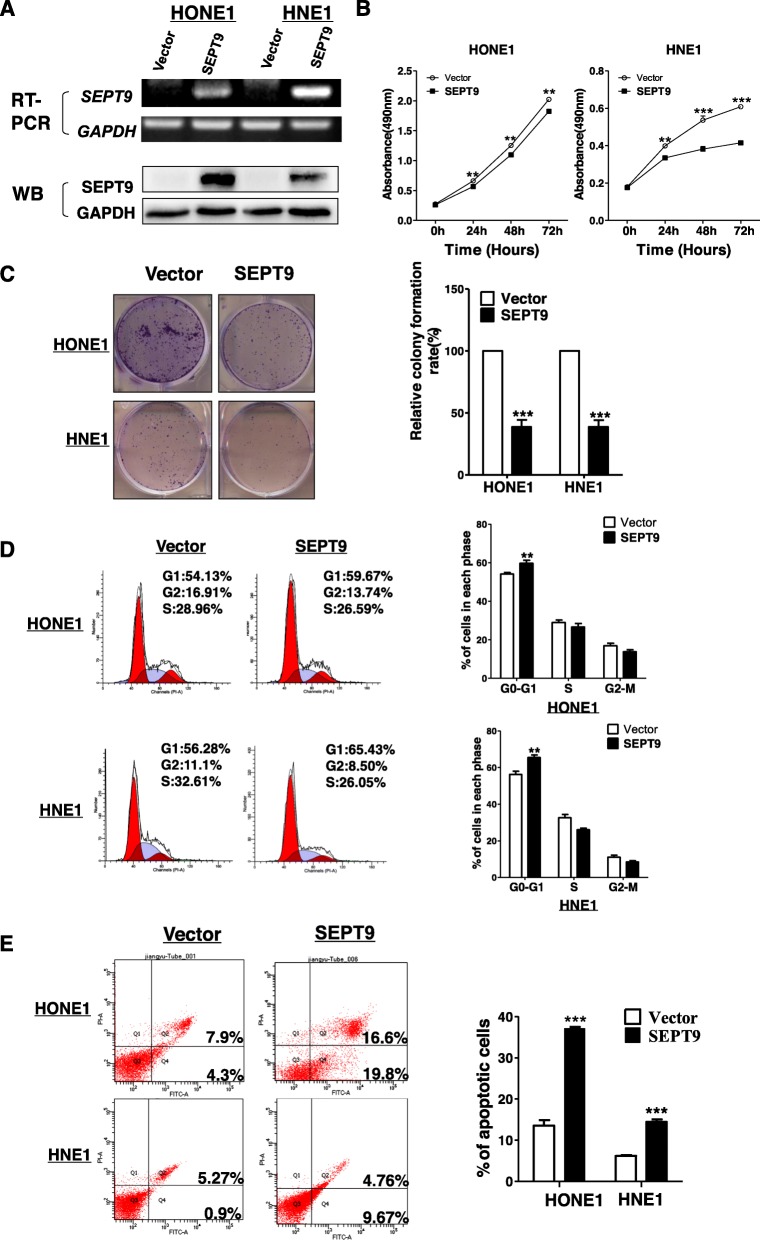
Ectopic SEPT9_v2 inhibited NPC cell proliferation and colony formation by inducing G0/G1 cell cycle arrest and apoptosis. HONE1 and HNE1 cells stably transfected with SEPT9_v2 or vector (pcDNA3.1) were used. **a** SEPT9_v2 mRNA expression was detected in HONE1 and HNE1 cells stably transfected with SEPT9_v2 or vector (pcDNA3.1) by RT-PCR and Western blot. FLAG-tag antibody was used to detect SEPT9_v2 protein. GAPDH was used as an input control. **b** The ability of cell proliferation was measured in stably transfected cells by MTS assay. **c** The stably transfected cells were used for colony formation assay to detect proliferation rates. Left: representative images. Right: histogram statistics of the colony formation assay. **d** The number of cells in the G0/G1 phase was detected in HONE1 and HNE1 cell lines stably transfected with SEPT9_v2 or vector. Left: representative flow cytometry plots. Right: histogram statistics of the distribution of cell population. **e** The proportion of apoptotic cells in transiently transfected HONE1 and HNE1 cell lines. Left: representative flow cytometry plots. Right: quantification of apoptosis changes. All values are presented as the mean ± SD of at least three independent experiments. SD, standard deviation. *p < 0.05, **p < 0.01, ***p < 0.001

Inhibition of SEPT9_v2 by promoter methylation in NPC suggests the loss of tumor-suppressive function. To assess this possibility, cell lines stably expressing SEPT9_v2 or vector as control were generated and confirmed by RT-PCR and Western blot assay (Fig. 2a). MTS and colony formation assays were performed to assess the effect of SEPT9_v2 on proliferation in NPC cell lines. Significantly reduced proliferation was observed in SEPT9_v2-expressing cells as evidenced by both assays (Fig. 2b, c). Since cell cycle arrest and apoptosis contribute to the inhibition of cell proliferation, flow cytometry was utilized to determine whether SEPT9_v2 overexpression affected NPC cell cycle arrest and/or apoptosis. The results showed that the number of cells in the G0/G1 phase was markedly increased in cells overexpressing SEPT9_v2, which was accompanied by a decrease in the G2/M and S phases in comparison with control cells (Fig. 2d). Furthermore, the number of apoptotic cells was significantly increased in cells expressing SEPT9_v2 compared to control (Fig. 2e). Hence, SEPT9_v2-induced NPC cell proliferation inhibition was associated with G0/G1 cell cycle arrest and apoptosis.

### SEPT9_v2 inhibits NPC cell migration and invasion

**Fig. 3:**
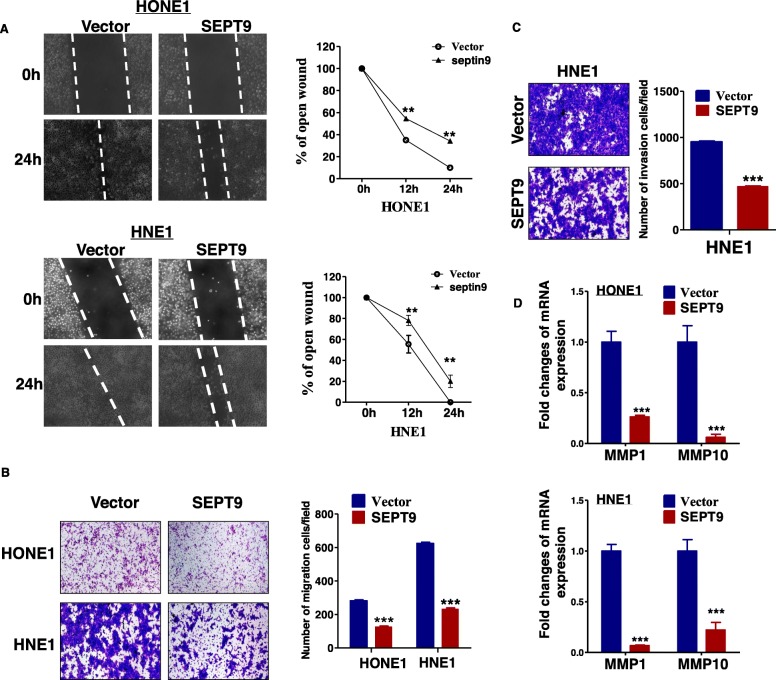
SEPT9_v2 inhibited NPC cell migration and invasion. **a** Stably transfected HONE1 and HNE1 cells in a wound healing assay at 0 and 24 h. Left: representative image. Right: distance of cell migration. **b**, **c** Transwell assay examined the migration and invasion of cells stably transfected with vector or SEPT9_v2. **d** The expression of MMP1 and MMP10 in the stably transfected cells detected by qPCR. MMP1, matrix metalloproteinase 1; MMP10, matrix metalloproteinase 10. All values are presented as the mean ± SD of at least three independent experiments. SD, standard deviation. *p < 0.05, **p < 0.01, ***p < 0.001

Effect of SEPT9_v2 expression on cell migration and invasion in NPC cell lines were assessed in wound healing assay and transwell assays. In wound healing assay, SEPT9_v2-transfected cells resulted in reduced relative migration over 24 h (Fig. 3a), and less SEPT9_v2-transfected cells passing through the membrane were observed in transwell assays, as compared to control cells, demonstrating SEPT9_v2 overexpression was associated with reduced capacity of migration and invasion in NPC (*p* < 0.001, Fig. 3b, c). Next, we examined the expression of matrix metalloproteinase 1 (MMP1) and matrix metalloproteinase 10 (MMP10), which are essential for cell migration and invasion, at the transcriptional levels. MMP1 and MMP10 expression were significantly downregulated in SEPT9_v2-overexpressing cells (Fig. 3d). Thus, SEPT9_v2 exerts a suppressive effect on migration and invasion in NPC cells.

### SEPT9_v2 inhibits proliferation and migration of NPC cells through inactivation of the Wnt/β-catenin signaling pathway via downregulation of FZD10

**Fig. 4:**
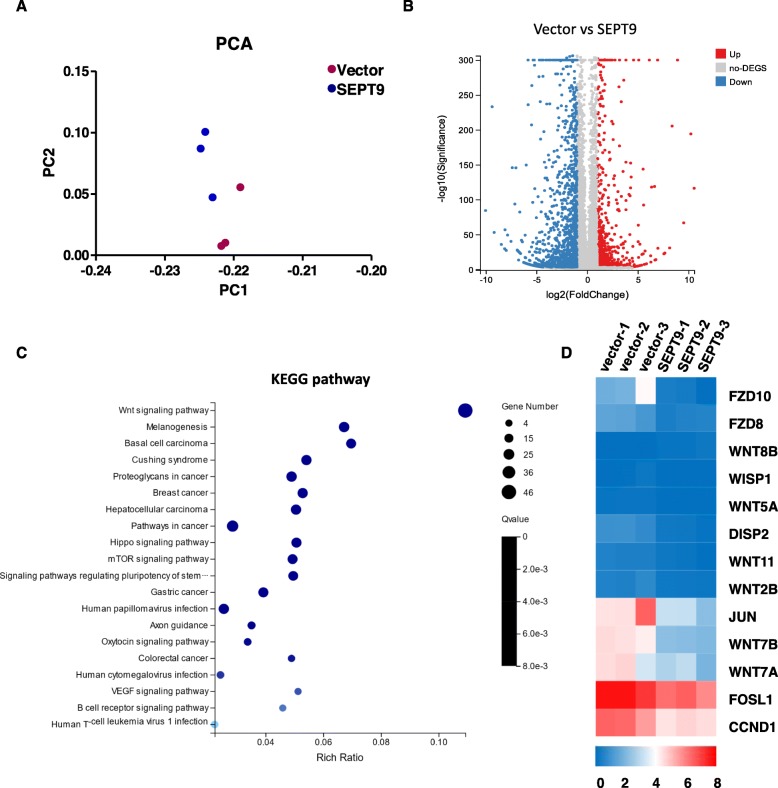
Analysis of RNA sequence. **a** The principal component analysis (PCA) of RNA-Seq. **b** Heat map analysis of differentially expressed genes in SEPT9_v2-transfected cells compared to empty vector control cells. **c** Wnt signaling pathway enrichment analyzed by the KEGG pathway enrichment. **d** The expression of a group of genes in the Wnt signaling pathway regulated by SEPT9_v2

**Fig. 5:**
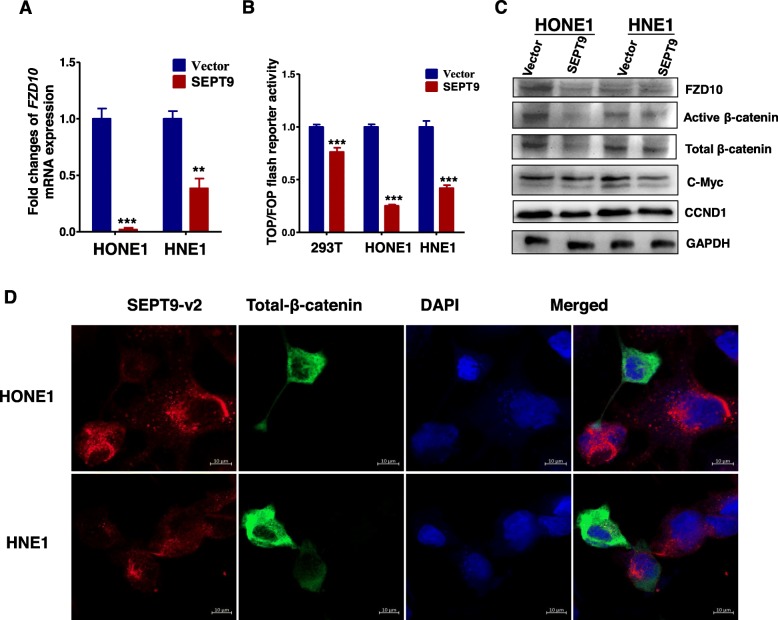
SEPT9_v2 inactivated the Wnt/β-catenin signaling pathway by downregulating FZD10. **a** FZD10 mRNA expression was detected by qPCR. Values are presented as the mean ± SD of at least three independent experiments. **b** TOP/FOP Flash reporter assays were performed to test the regulation of SEPT9_v2 on the Wnt/β-catenin pathway in stably transfected HONE1 cells, HNE1 cells, and 293T cells. Values are presented as the mean ± SD of at least three independent experiments. **c** Western blots were performed to detected β-catenin and the downstream Wnt/β-catenin genes. **d** The expression of SEPT9_v2 and β-catenin were detected by immunofluorescence staining in stably transfected HONE1 and HNE1 cells. SD, standard deviation. The nuclei were counterstained with DAPI. DAPI, 4,6-diamidino-2-phenylindole. *p < 0.05, **p < 0.01, ***p < 0.001

**Fig. 6:**
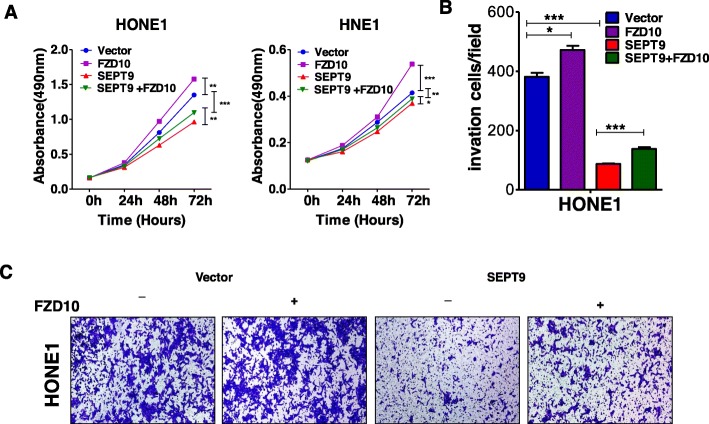
FZD10 plasmid attenuated SEPT9_v2 transfection effects in HONE1 and HNE1 cells, modifying proliferation and migration. **a**, **b** Effect of FZD10 overexpression on proliferation in HONE1 and HNE1 cells by MTS assay. **c** Effect of FZD10 overexpression on migration in HONE1 cells by transwell migration assay. All values are presented as the mean ± SD of at least three independent experiments. SD, standard deviation. *p < 0.05, **p < 0.01, ***p < 0.001

RNA sequence was performed to assess SEPT9_v2 target genes in HONE1 cells after stable transfection of SEPT9_v2-expressing pcDNA3.1 plasmid or an empty vector. Figure 4a shows the principal component analysis (PCA) in more detail. SEPT9_v2-transfected cells showed upregulation of 685 genes and downregulation of 1474 genes compared to empty vector control cells (Fig. 4b). To define crucial pathways regulated by SEPT9_v2, the Kyoto Encyclopedia of Genes and Genomes (KEGG) pathway enrichment analysis was performed [24]. These upregulated genes and downregulated genes were classified into different KEGG pathways. Among the pathways, the Wnt signaling pathway was pinpointed down as its crucial role in tumor proliferation and cancer development (Fig. 4c). Noteworthily, SEPT9_v2 significantly repressed the expression of a group of genes in the Wnt signaling pathway (Fig. 4d), and among which, FZD10, a seven-transmembrane receptor of the Wnt/β-catenin pathway, disclosed a 5.19-fold reduction. Other FZD receptors for the Wnt signaling pathway were unaffected or slightly affected after SEPT9_v2 transfection. To determine whether SEPT9_v2 reduced the activation of the Wnt pathway via FZD10, qPCR was performed. The results showed ectopic expression of SEPT9_v2 in NPC cells resulted in decreased FZD10 mRNA expression (Fig. 5a). As shown in TOP/FOP Flash reporter assays performed in 293T, HONE1, and HNE1 cells, ectopic SEPT9_v2 expression markedly inhibited TCF/LEF luciferase activity, which is a hallmark of canonical Wnt activation (Fig. 5b). Furthermore, Western blot assay revealed that FZD10 and target proteins of Wnt/β-catenin including total β-catenin, active β-catenin, c-Myc, and cyclinD1 were downregulated (Fig. 5c). Similarly, SEPT9_v2 overexpression markedly decreased β-catenin in HONE1 and HNE1 as confirmed by immunofluorescence staining (Fig. 5d). Rescue experiments were further performed to examine the effects of SEPT9_v2/FZD10 on proliferation and migration in NPC. The data showed that co-transfection with an FZD10 plasmid effectively attenuated SEPT9_v2 transfection effects in NPC cells, modifying proliferation and migration (Fig. 6a–c).

### SEPT9_v2-induced suppression of FZD10 mRNA may be mediated by miR-92b-3p

**Fig. 7:**
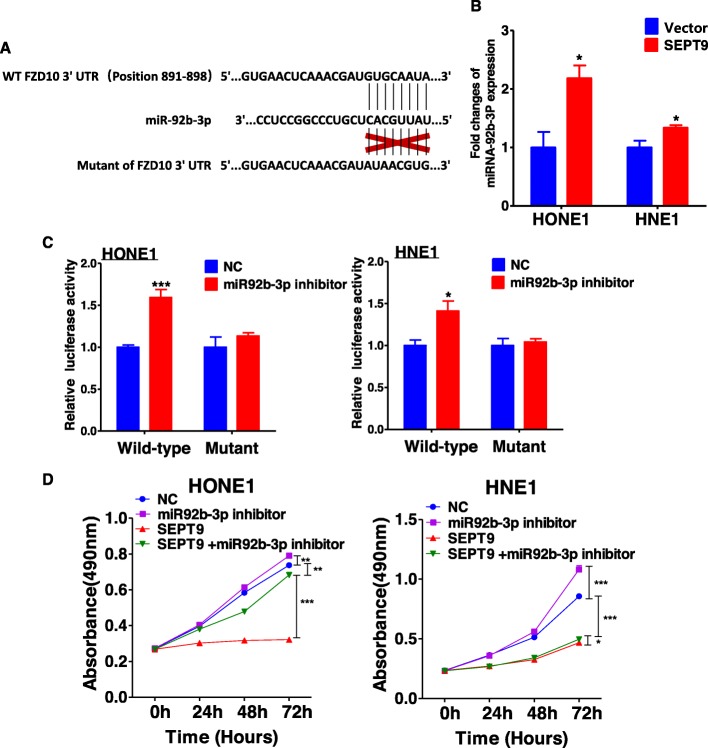
SEPT9_v2 decreased the expression of FZD10 through altering the miR-92b-3p level. **a** The duplex formation between human wild-type (WT) FZD10 3′-UTR and has-miR-92b-3p predicted from the TargetScan. **b** miR-92b-3p expression level was detected in stably transfected cell lines by qPCR. **c** The regulation of miR-92b-3p on WT and MT of FZD10 3′-UTR was detected by luciferase assay in HONE1 and HNE1 cells transfected with miR-NC or miR-92b-3p inhibitor oligo. **d** Effect of miR-92b-3p inhibitor transfection on proliferation in HONE1 and HNE1 cells via MTS assay. All values are presented as the mean ± SD of at least three independent experiments. SD, standard deviation. *p < 0.05, **p < 0.01, ***p < 0.001

It is known that microRNAs can mediate gene silencing by targeting 3′-UTRs of mRNAs. The overall downregulation of miRNA expression is a feature of cancer, and some microRNAs are prognostic markers for cancer [25]. We investigated whether microRNAs are involved in the regulation of FZD10 mRNA expression by SEPT9_v2. Bioinformatic analysis (TargetScan) [26] was used to search potential microRNAs and corresponding binding sites within the 3′-UTR that could regulate FZD10 (Fig. 7a). Among the microRNAs retrieved from TargetScan, the expression of miR-92b-3p was upregulated in SEPT9_v2-overexpressing NPC cells (Fig. 7b). To determine whether miR-92b-3p could target the 3′-UTR of FZD10 mRNA to directly suppress FZD10, a pmiR-RB-Report™ vector carrying mutant-type (MT) or wild-type (WT) 3′-UTR sequences of FZD10 was assessed by luciferase reporter assay. a pmiR-RB-Report™ vector carrying mutant-type (MT) 3′-UTR sequences of FZD10 was used as a control. miR-92b-3p was found to inhibit luciferase activity of WT 3′-UTR with no obvious effect on the MT 3′-UTR reporter (Fig. 7c). These results indicate that SEPT9_v2 can inhibit FZD10 via upregulation of miR-92b-3p. We further investigated whether the effect of the SEPT9_v2-miR-92b-3p-FZD10 signaling axis on cell proliferation of NPC was sustained with the miR-92b-3p inhibitor. MTS assay was performed, and the results confirmed that co-transfection with miR92b-3p inhibitor partially reversed the effect of SEPT9_v2 on cell proliferation and promoted the proliferation in NPC cells (Fig. 7d). Hence, these results above indicated that SEPT9_v2-induced suppression of FZD10 mRNA may be mediated by miR-92b-3p.

## Discussion

As the most complex member of the septin family of genes, SEPT9 plays a crucial role in tumorigenesis. However, whether SEPT9 is an oncogene or a tumor suppressor is controversial in that both pro- and anti-oncogenic properties have been described. Sept9_v1 and Sept9_v3 expression levels have been found to be increased in breast tumors when compared to expression levels within matched normal tissues. Further, SEPT9_v1 can accelerate growth kinetics, stimulate cell motility, and promote invasion [14, 27, 28]. Conversely, Sept9_i2 is downregulated in breast tumors and can inhibit the migration of cancer cells [29]. These observations suggest that specific SEPT9 variants have different effects on oncogenesis.

In this study, SEPT9_v2 expression was shown to be downregulated in NPC cell lines and tissues by methylation of the promoter region. However, Fig. 1b shows the overlap between NPC and the control group because the samples are not paired. In addition, the number of samples in each group is relatively low. These are limitations of our study. In future studies, more paired samples will be collected to give the statement more credence. Ectopic expression of SEPT9_v2 inhibited NPC cell proliferation by inducing cell cycle arrest in the G0/G1 phase as well as by promotion of apoptosis. Overexpression of Sept9_v2 was shown to inhibit NPC cell migration and invasion. Therefore, SEPT9_v2 appears to act as a suppressor of tumorigenesis. Further, functional loss of SEPT9_v2 may be a causative event in the pathogenesis of NPC.

Aberrant expression of the Wnt pathway constituents can induce diseases, particularly cancer [30]. A variety of genes regulate NPC progression via the Wnt pathway. For example, SOX1 downregulates β-catenin and reverses the malignant phenotype of NPC [31]. MicroRNA-506 inhibits NPC tumor growth and metastasis through the inactivation of the Wnt/β-catenin signaling pathway [32]. YPEL3 suppresses NPC epithelial to mesenchymal transition and metastasis by suppressing the Wnt/β-catenin signaling pathway [33]. We focused on FZD10 because, as a receptor for the canonical Wnt pathway, it is known to enhance oncogenic Wnt signaling as well as to regulate cell function in different cancers [34–39]. In addition, RNA sequence data showed a significant downregulation of FZD10 mRNA levels after SEPT9_v2 transfection. Hence, FZD10 appears to play a key role in SEPT9_v2-mediated inactivation of the Wnt signaling pathway. As expected, the data validated the downregulation of Wnt target proteins with inhibition of TCF/LEF luciferase activity as judged by Western blot, immunofluorescence, and luciferase assay. The effect of FZD10 on NPC cell proliferation and migration was also validated. These results suggested SEPT9_v2 inhibits proliferation and migration of NPC cells through the inactivation of the Wnt/β-catenin signaling pathway by downregulation of FZD10.

Since microRNAs play crucial roles in virtually every cellular process, especially tumorigenesis and development, we evaluated the involvement of microRNAs. miR-92b has been shown to target many genes and to promote cell proliferation and invasiveness of lung cancer, bladder cancer, and oral squamous cell carcinoma [40–42]. For pancreatic cancer, miR-92b-3p acts as a tumor suppressor by targeting Gabra3 [43]. In NPC, miR-92b inhibits migration and invasion by targeting Smad3 [44]. Although the effect of miR-92b-3p on FZD10 expression showed a limited effect size, we can still speculate that miR-92b-3p may inhibit the canonical Wnt/β-catenin pathway via targeting FZD10. Hence, SEPT9_v2 may serve to upregulate miR-92b-3p with the targeting of the 3′-UTR of FZD10.

Further work will be focused on investigating how could SEPT9_v2 upregulate miR-92b-3p and downregulate FZD10. We consider it a meaningful study to reveal the exact mechanism between them. A research has provided us some enlightenment. This research has revealed that interaction with septin complex may enhance the accessibility of HDAC6 to acetylated α-tubulin, which plays a crucial role in the regulation of gene transcription [45].

## Conclusions

**Fig. 8:**
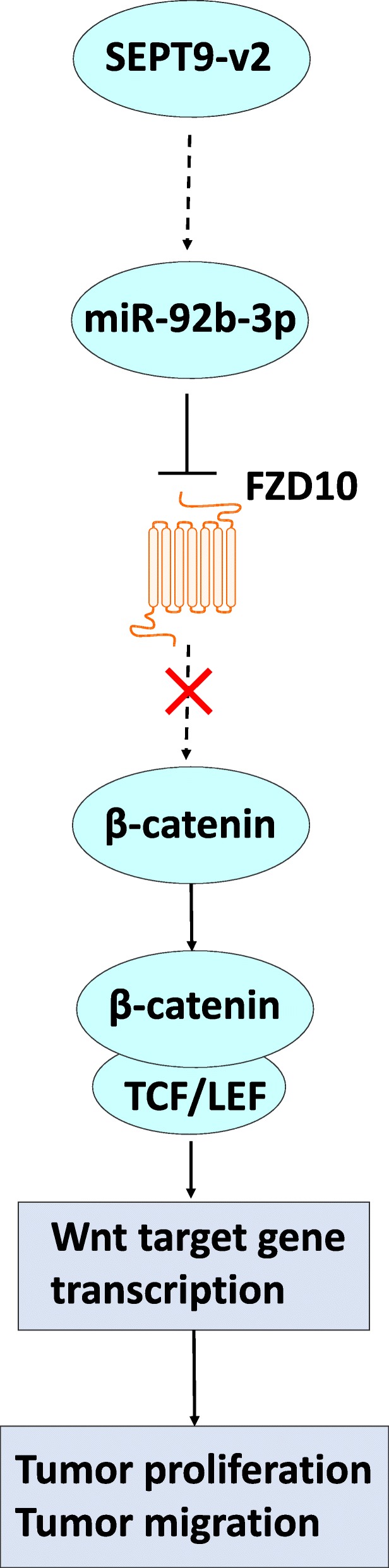
Schematic summary of the SEPT9_v2/miR92b-3p/FZD10 axis

In summary, promoter hypermethylation downregulates SEPT9_v2, which acts as a NPC tumor suppressor through inactivation of the Wnt/β-catenin signaling pathway via miR92b-3p/FZD10 (Fig. 8). This study suggests that SEPT9_v2 may be a promising therapeutic target and biomarker for NPC and therefore worthy of further study in the future.

## Methods

### Tumor cell lines and tissues

Two poorly differentiated human NPC cell lines (HONE1 and HNE1, Chinese University of Hong Kong, Hong Kong, China) were used in this study. These cell lines were both taken from poorly differentiated squamous cell carcinomas of the nasopharynx. HONE1 was obtained from a 68-year-old Chinese man. HNE1 cell line was obtained from a 27-year-old Chinese man. The HONE1 cell line has been passaged more than 90 times and the HNE1 cells more than 100 times [46]. HONE1 and HNE1 were cultured in Roswell Park Memorial Institute (RPMI) 1640 (Gibco BRL, MD, USA) supplemented with 10% fetal bovine serum (FBS, Gibco, CA, USA) and 1% penicillin and streptomycin (Gibco) at 37 °C with 5% CO_2_. PcDNA3.1 or pcDNA3.1–SEPT9_v2-FLAG-tag plasmids (4 μg each) were transfected into HONE1 and HNE1 cells using Lipofectamine 2000 (Invitrogen) to construct stable cell lines based on the manufacturer’s instructions. Forty-eight hours after transfection, cells were selected by G418(50 mg/mL) with a concentration of 8 μL/mL (HONE1) and 6 μL/mL (HNE1) for more than 14 days. The expression of SEPT9_v2 in the transfected cells was validated by RT-PCR and Western blot. FLAG-tag antibody was used to detect SEPT9_v2 protein. PcDNA3.1 plasmid was used as a control. Tissues were obtained from patients at the Department of Otolaryngology of the First Affiliated Hospital of Chongqing Medical University previously [47, 48]. All samples were evaluated histologically. All patients provided written informed consent before enrollment. The study was performed with the approval of the Ethics Committees of the First Affiliated Hospital of Chongqing Medical University (approval notice #20130306).

### RNA isolation, RT-PCR, semi-quantitative PCR, and qPCR

Total RNA was extracted from cell lines and tissues with TRIzol Reagent (Invitrogen, Carlsbad, CA, USA), according to the protocols recommended by the manufacturer. Extracted total RNA was quantified using spectrophotometry analysis and reserved at − 80 °C until use. RT-PCR was performed using Go-Taq (Promega, Madison, WI, USA) for 32 cycles of amplification with GAPDH as a control. The primer sequences are listed in Table 2. Go-Taq (Promega) and 2% agarose gels were sequentially used to perform PCR and gel electrophoresis (120 V, 25 min) according to the standard procedure. A BioRad Gel Doc XR+ system was used to analyze the results. SYBR Green (Thermo Fisher Scientific, Hong Kong, China) was used to perform qPCR, according to the manufacturer’s instructions using a 7500 Real-Time PCR System (Applied Biosystems, Foster City, CA, USA). GAPDH was amplified as control. Gene expression levels were calculated by the 2−ΔΔCt method. All samples were assessed in triplicate.

**Table 2:** List of primers used in this study

PCR	Primer	Sequence (5′-3′)	Product size (bp)	PCR cycles	Annealing temperature (°C)
qRT-PCR	GAPDHF	GGAGTCAACGGATTTGGT	206	23	55
	GAPDHR	GTGATGGGATTTCCATTGAT			
	SEPT9_v2F	TGGGATCATTTCGGACTTCG	167		60
	SEPT9_v2R	AGGGTTCTGAGTTCTTCACG			
	MMP1F	GAGATCATCGGGACAACTCTCCTT	119		
	MMP1R	GTTGGTCCACCTTTCATCTTCATCA			
	MMP10F	AGACACTGACACTCTGGAGG	202		
	MMP10R	TGGAGTCACCTCTTCCCAGA			
	FZD10F	ATCGGCACTTCCTTCATCCTGTC	199		
	FZD10R	TCTTCCAGTAGTCCATGTTGAG			
	miR-92b-3pF	TATTGCACTCGTCCCG			
	miR-92b-3PR	GTGCAGGGTCCGAGGT			
	miR-92b-3PRT	GTCGTATCCAGTGCAGGGTCCGAGGTATTCGCACTGGATACGACGGAGGC			
	U6F	CTCGCTTCGGCAGCACA			
	U6R	AACGCTTCACGAATTTGCGT			
	U6RT	AACGCTTCACGAATTTGCGT			
	GAPDHF	CCAGCAAGAGCACAAGAGGAA	114		
	GAPDHR	CAAGGGGTCTACATGGCAACT			
MSP	SEPT9_v2m1	GGTTTAGGGGTTTTTTCGGC	137		60
	SEPT9_v2m2	AACTAATAAACAACGAATCGCG			
	SEPT9_v2u1	GGGTTTAGGGGTTTTTTTGGT	141		58
	SEPT9_v2u2	AATAACTAATAAACAACAAATCACA			

### Aza and TSA treatment, DNA isolation, and MSP

HONE1 and HNE1 were treated with 10 μmol/L DNA methyltransferase (DNMT) inhibitor (Aza, Sigma-Aldrich, Steinheim, Germany) for 72 h and treated or not with 100 nmol/L trichostatin A (TSA; Cayman Chemical Co., Ann Arbor, MI) for 24 h. RNA was extracted for qPCR. Genomic DNA was isolated from tissues and cell lines using a QIAamp DNA Mini kit (Qiagen, Hilden, Germany), according to the manufacturer’s instructions. Extracted DNA was quantified using spectrophotometry analysis and reserved at − 20 °C until use. AmpliTaq-Gold DNA Polymerase (Applied Biosystems) was used to perform MSP, with annealing temperatures at 60 °C for methylated samples and 58 °C for unmethylated samples (Table 2). MSP primers for SEPT9_v2 are shown in Table 2. The primers were all previously determined to not amplify non-bisulfinated DNA. PCR products were assessed with 2% agarose gels.

### miRNA inhibitor and transfection

The miR-NC (negative control) and miR-92b-3p inhibitor were synthesized by RIBOBIO (Guangzhou Ribobio Co., Ltd). A concentration of 75 nM miR-92b-3p inhibitor or miR-NC per well were transfected using Lipofectamine 2000 according to the manufacturer’s instructions. Forty-eight hours after transfection, RNA was extracted for quantitative real-time PCR (qPCR) analysis.

### Western blot assay

Radioimmunoprecipitation assay (RIPA) buffer with a phosphatase inhibitor cocktail and a protease inhibitor (Sigma, St. Louis, MO, USA) was used to isolate proteins from cells. The protein concentration was measured using the bicinchoninic acid (BCA) assay (Thermo Scientific, Rockford, IL, USA). Standard Western blot techniques were performed as previously described [49]. The antibodies used were GAPDH (1:1000) (#2118, Cell Signaling Technology, Danvers, MA, USA), FLAG-tag (1:1000) (#14793, Cell Signaling Technology), FZD10 (1:500) (41686, Signalway Antibody, MD, USA), total β-catenin (1:1000) (#2677, Cell Signaling Technology), active β-catenin (1:1000) (#19807s, Cell Signaling Technology), CCND1 (1:1000) (cyclinD1, #1677-1, Epitomics), c-Myc (1:1000) (#13987s, Cell Signaling Technology).

### Cell proliferation assay and colony formation assay

For cell proliferation assay, stably transfected HONE1 and HNE1 cells were cultured in 96-well plates at a density of 2000 cells per well. Cell proliferation assay (MTS, Promega) was used to measure cell viability at 0, 24, 48, and 72 h according to the manufacturer’s protocol. Cells were incubated at each time point with 100 μL medium and 20 μL CellTiter 96 Aqueous One Solution reagent at 37 °C for 2 h. Absorbance was read at 490 nm with a microplate reader (Multiskan MK3, Thermo Fisher Scientific, Schwerte, Germany). For colony formation assay, stably transfected HONE1 and HNE1 cells were plated in 6-well plates at a density of 200, 400, and 800 cells per well and then cultured for approximately 10 days until colonies were visible. The surviving colonies (≥ 50 cells/colony) were counted after fixation in formaldehyde and 10% gentian violet staining.

### Flow cytometric analysis of cell cycle and apoptosis

Cell cycle distribution and the percentage of apoptotic cells were assessed by Flow cytometry. For cell cycle analysis, stably transfected HONE1 and HNE1 cells were collected and washed with PBS, fixed in ice-cold 70% ethanol overnight at − 4 °C, then treated with RNase A (Sigma) at a concentration of 5 mg/mL, and stained with propidium iodide (PI; BD Pharmingen, San Jose, CA, USA) (10 mg/mL in PBS) for 30 min at room temperature in the dark. For apoptosis, stably transfected cells were collected and stained with annexin V-fluorescein isothiocyanate and PI according to the manufacturer’s instructions. Data were analyzed with the CELL Quest software (BD Biosciences, San Jose, CA).

### Wound healing, transwell migration, and invasion experiments

For the wound healing assay, HONE1 and HNE1 cells were stably transfected with SEPT9_v2 or vector plasmid and then placed in 6-well plates until confluent. Distance of cell migration was imaged at 0, 12, and 24 h. For the transwell assays, chambers (Corning Life Sciences, Corning, NY, USA) with an 8-μm diameter were covered with or without Matrigel (2.5 mg/mL) (BD Biosciences) to evaluate migration or invasion, respectively. Cells were suspended in RPMI 1640(-) and seeded to the upper well of the chambers. RPMI 1640 containing 20% FBS was added to the lower chamber well and incubated at 37 °C for 30 h. The cells passing through the chamber and remaining in the lower side of the membrane were fixed with 4% paraformaldehyde for 30 min and then stained with crystal violet for 20 min. Three random fields were selected for photography (Leica DMI4000B; Leica Microsystemes, Milton Keynes, Buckinghamshire, UK).

### Immunofluorescence assay

To detect the effect of SEPT9_v2 on β-catenin, an immunofluorescence assay was performed with HONE1 and HNE1 cells. The cells were seeded onto slides in 24-well plates and then transfected with pcDNA3.1-SEPT9_v2-FLAG-tag or pcDNA3.1 plasmid. After 48 h, cells were fixed in 4% paraformaldehyde for 30 min and then permeabilized with 0.5% Triton X-100 for 10 min and blocked with blocking buffer for 1 h. Next, antibodies against β-catenin (1:500) (#2677, Cell Signaling Technology) and FLAG-tag (1:500) (#14793, Cell Signaling Technology) were used to stain the cells overnight at 4 °C, followed by incubation with Alexa Fluor 488- or 594-conjugated goat anti-mouse or anti-rabbit secondary antibody (Jackson ImmunoResearch, West Grove, PA, USA) for 2 h. The nuclei were counterstained with 4,6-diamidino-2-phenylindole (DAPI, Roche, Palo Alto, CA, USA) and imaged using a confocal laser scanning microscope.

### Dual-luciferase reporter assay

To explore the effect of SEPT9_v2 on T cell factor/lymphoid enhancer factor (TCF/LEF) transcriptional activities, TOPflash luciferase reporter plasmids containing TCF/LEF binding sites and FOPflash luciferase reporter plasmids containing a mutant TCF/LEF binding site were assayed. Cells were seeded into 24-well plates and were transiently co-transfected with TOPflash or FOPflash luciferase reporter plasmids and SEPT9_v2 or vector (pcDNA3.1). Renilla luciferase reporter pRL-TK (Promega) was added to each well as an internal control. After 48 h, a dual-luciferase reporter assay kit (Promega) was used to detect luciferase activity according to the manufacturer’s instructions. Firefly luciferase activity was normalized to *Renilla* luciferase activity and presented as a relative activity. To verify FZD10 as a direct target of miR-92b-3p, luciferase reporter plasmids (Guangzhou Ribobio Co, Ltd, Guangzhou, China) containing WT or MT FZD10 3′-UTR (3′-untranslated region) were assessed. Cells were seeded into 24-well plates and were transiently co-transfected with WT or MT FZD10 3′-UTR reporter plasmids and SEPT9_v2 or vector (pcDNA3.1). Renilla luciferase reporter pRL-TK (Promega) was added to each well as an internal control. The follow-up steps were performed as described above.

### RNA sequence analysis

To explore the target genes modulated by SEPT9_v2, RNA sequence analysis was performed in HONE1 cells stably transfected with either pcDNA3.1-SEPT9_v2 or an empty vector as control. The assay was performed by the BGI tech company (BGI-Shenzhen, china). The results were analyzed by the system provided by the company. The procedure of mRNA library construction was approximately as follows: mRNA was extracted and purified by oligo-dT beads and then fragmented into small pieces with fragment buffer. First-strand or second-strand cDNA were generated in the strand reaction system by PCR. Then, magnetic beads were used to purify the reaction product. A-Tailing Mix was added to incubate 30 min at 20 °C, and then RNA index adapters were added to end repair. The products were amplified and then purified by Ampure XP Beads. The library was validated by the 2011 bioanalyzer for quality control and then undergoes DSN treatment. To ensure the high quality of data, the distribution of the fragment size was checked by two methods: Agilent 2100 bioanalyzer or ABI StepOnePlus Real-Time PCR System (TaqMan Probe). The qualified library from the previous step was amplified on cBot. The final library was sequenced single end on the X-ten platform or HiSeq4000.

### Statistical analysis

GraphPad Prism 5.0 software and IBM SPSS 22.0 software was used to analyze the data. Two-tailed Student’s *t* tests, Mann-Whitney *U* test, and the *χ*^2^ test were used to assess *p* values with a *p* < 0.05 considered statistically significant. All values are presented as the mean ± SD of at least three independent experiments.

## Supplementary information

**Additional file 1: Figure S1.** SEPT9_v1 and SEPT9_v3-7 promoter methylation in paired or independent HNSC and HNSN tissue samples or from MethHC database. (A) SEPT9_v1 promoter methylation in paired or independent HNSC and HNSN tissue samples. (B) SEPT9_v3 promoter methylation in paired or independent HNSC and HNSN tissue samples. (C) SEPT9_v4 promoter methylation in paired or independent HNSC and HNSN tissue samples. (D) SEPT9_v5 promoter methylation in paired or independent HNSC and HNSN tissue samples. (E) SEPT9_v6 promoter methylation in paired or independent HNSC and HNSN tissue samples. (F) SEPT9_v7 promoter methylation in paired or independent HNSC and HNSN tissue samples.

## Data Availability

The datasets used and/or analyzed during the current study are available from the corresponding author on reasonable request.

## Abbreviations

Aza: 5-Aza-2′-deoxycytidine
DAPI: 4,6-Diamidino-2-phenylindole
DNMT: DNA methyltransferase
FBS: Fetal bovine serum
FZD10: Frizzled class receptor 10
HNSC: Head and neck squamous cell carcinomas
HNSN: Head and neck squamous epithelium
KEGG: Kyoto Encyclopedia of Genes and Genomes
M: Methylated
MMP1: Matrix metalloproteinase 1
MMP10: Matrix metalloproteinase 10
MSP: Methylation-specific PCR
MT: Mutant-type
NM: Normal nasal mucosal
NPC: Nasopharyngeal carcinoma
PCA: Principal component analysis
PI: Propidium iodide
qPCR: Quantitative reverse transcription polymerase chain reaction.
RPMI: Roswell Park Memorial Institute
RT-PCR: Reverse transcription polymerase chain reaction
U: Unmethylated
WT: Wild-type
