# Supplementary figures and images for: SEPT9_v2, frequently silenced by promoter hypermethylation, exerts anti-tumor functions through inactivation of Wnt/β-catenin signaling pathway via miR92b-3p/FZD10 in nasopharyngeal carcinoma cells
Source: Clin Epigenetics. 2020 Mar 5;12:41. doi: 10.1186/s13148-020-00833-5 (PMC7059696; doi:10.1186/s13148-020-00833-5)

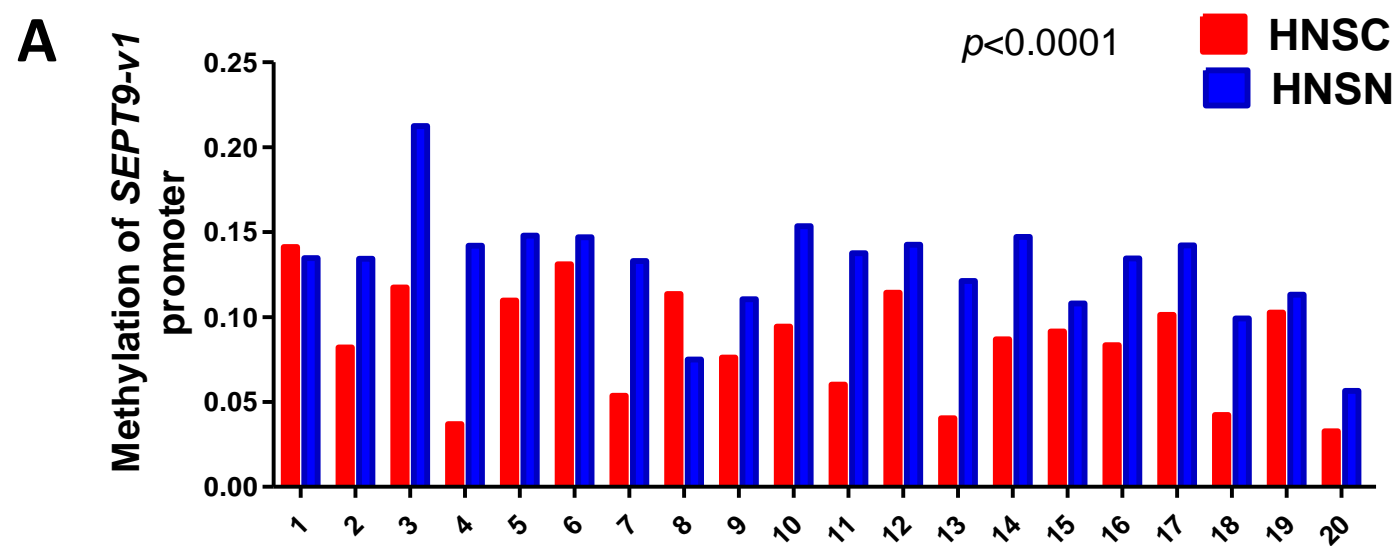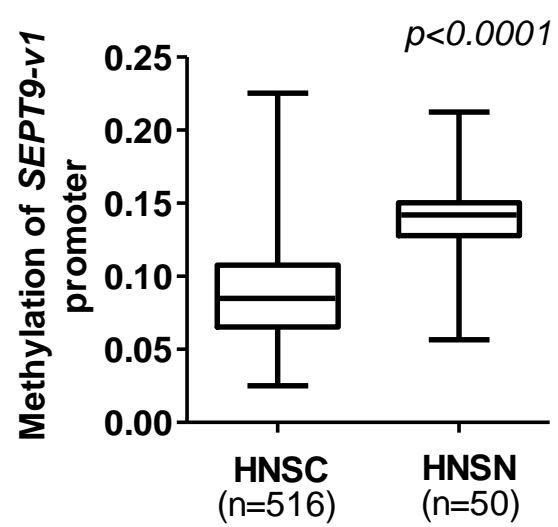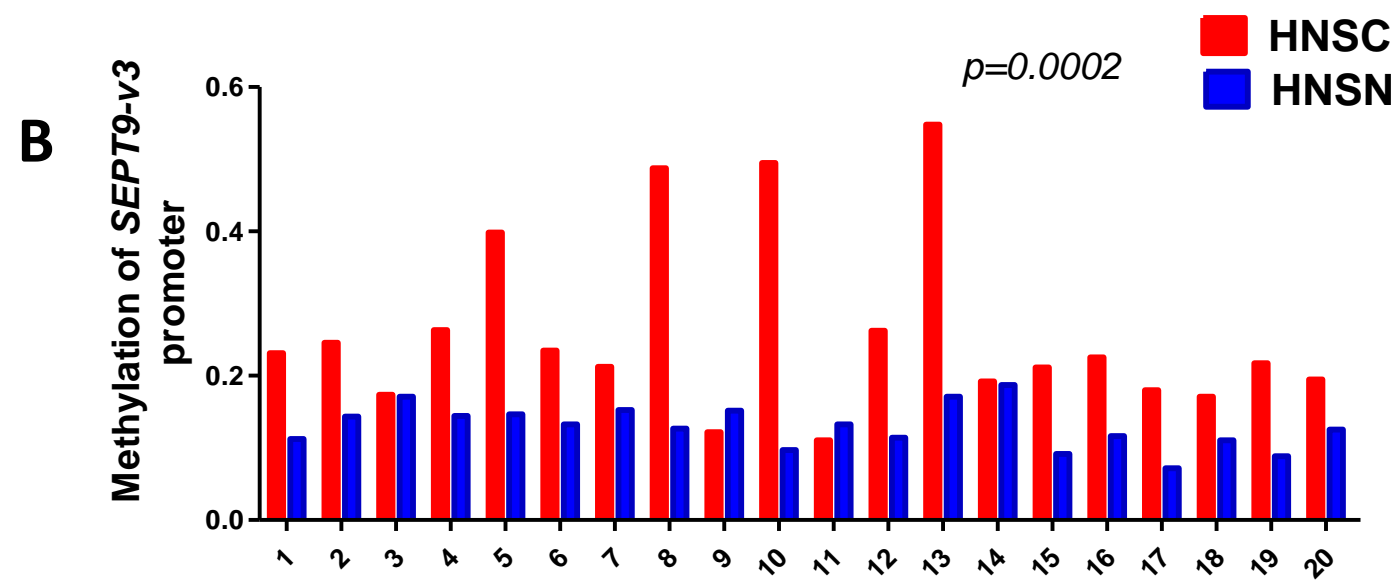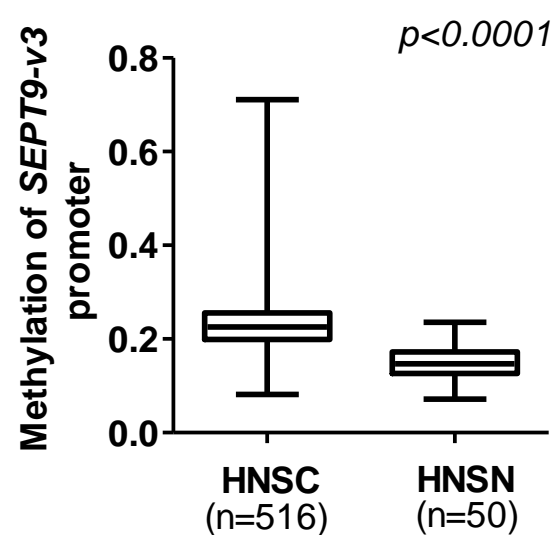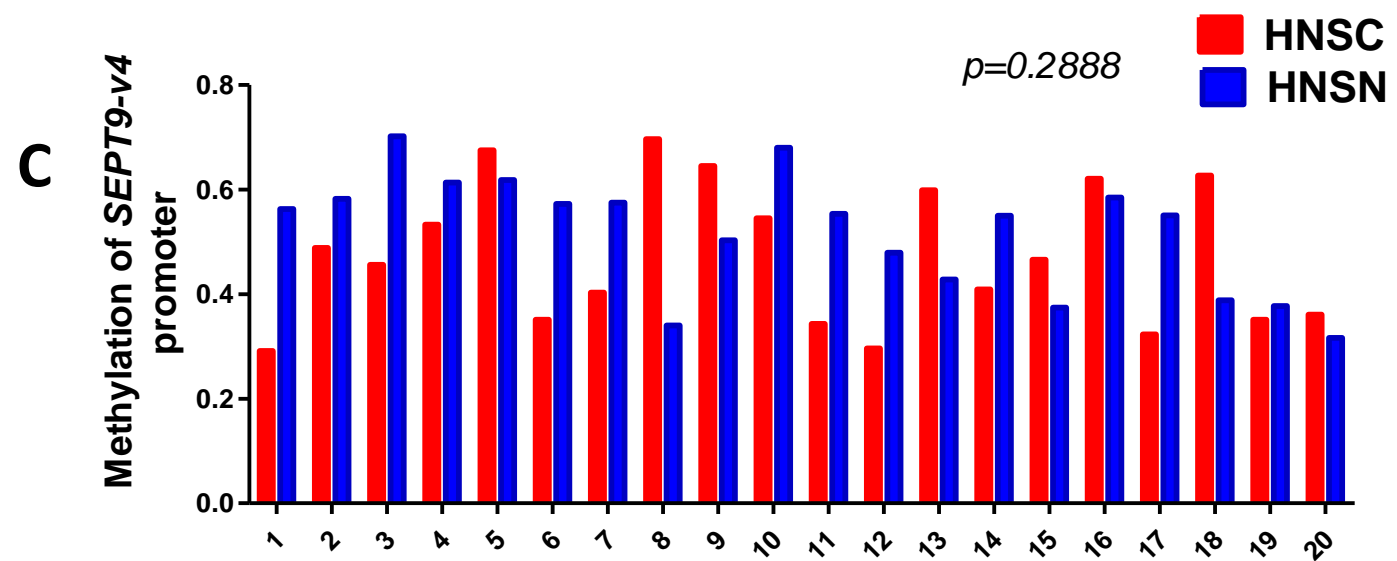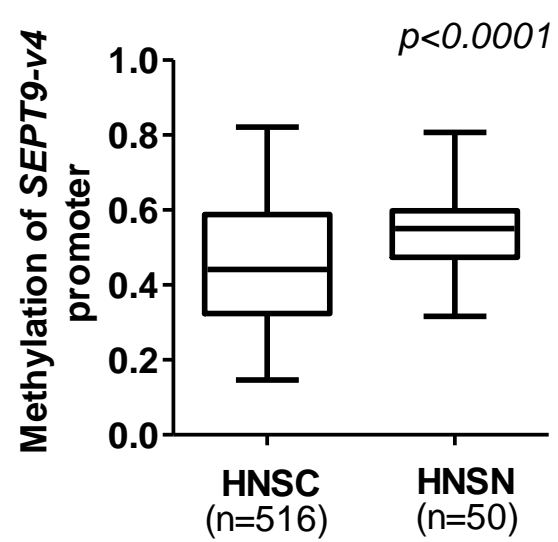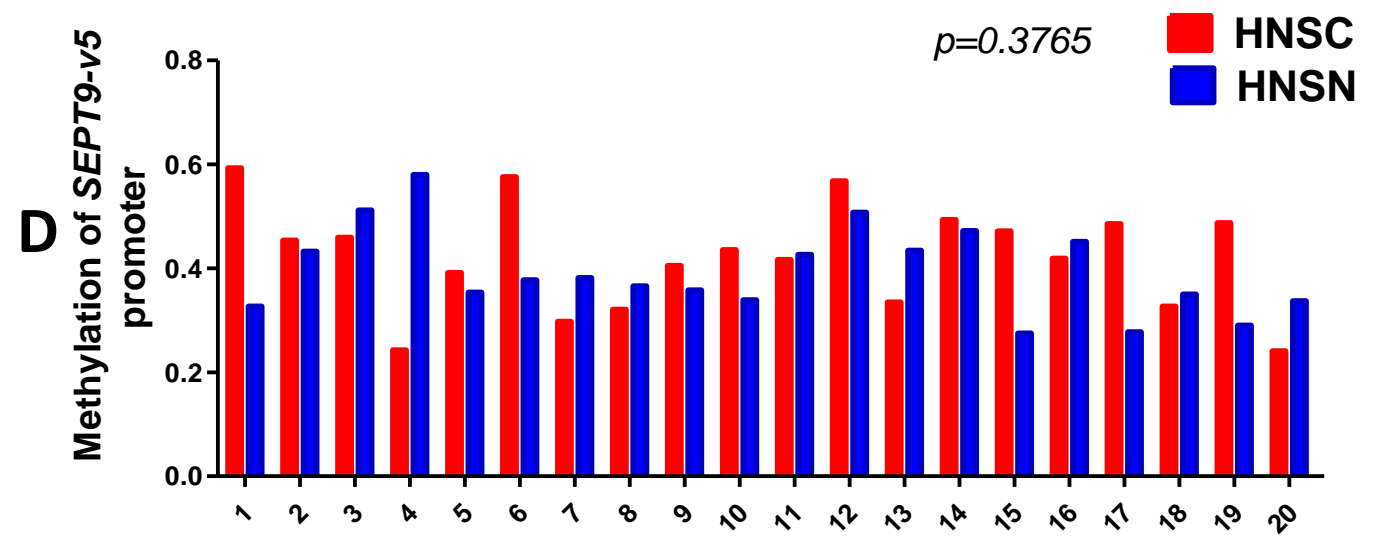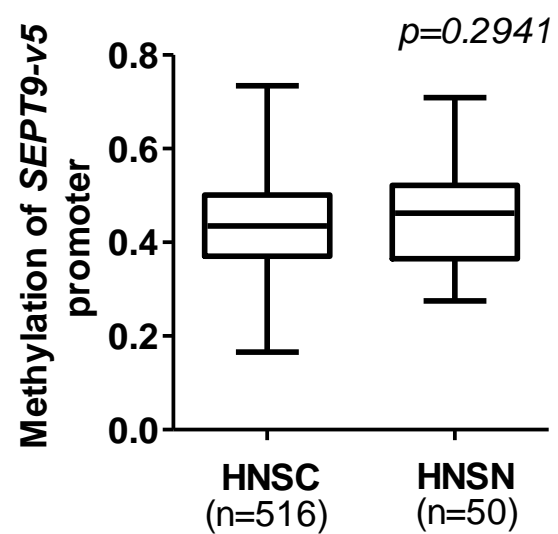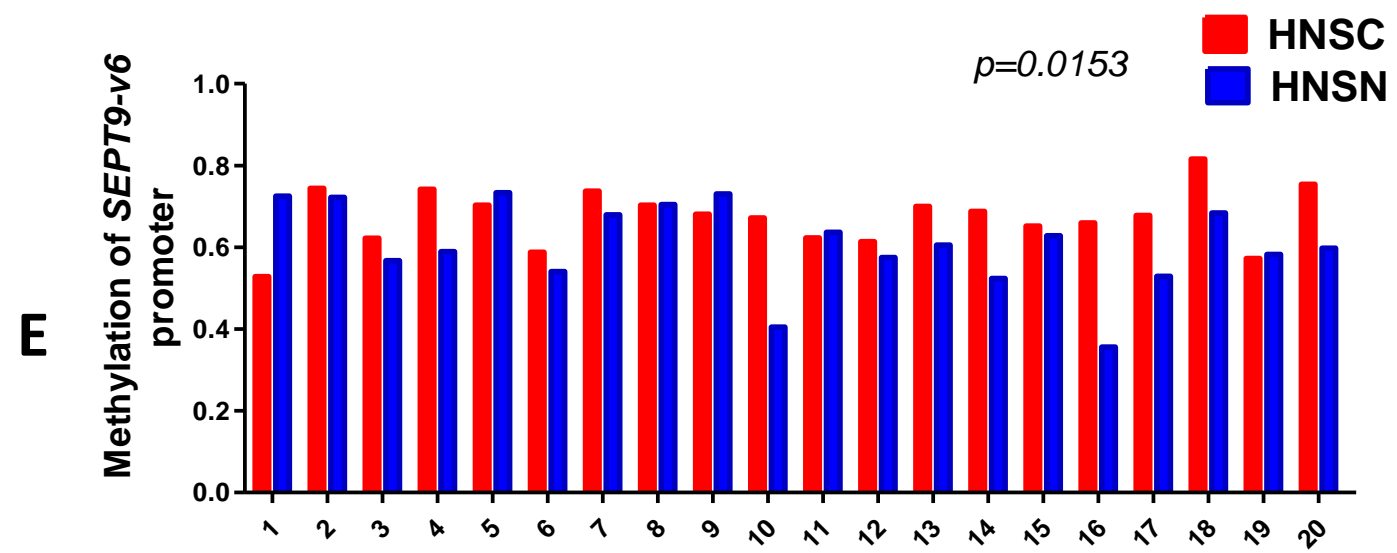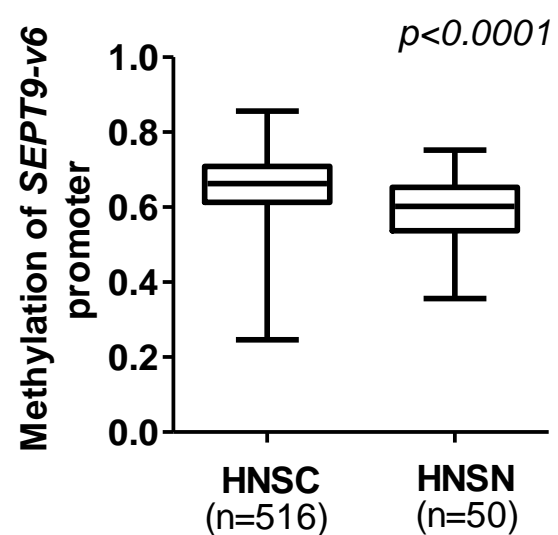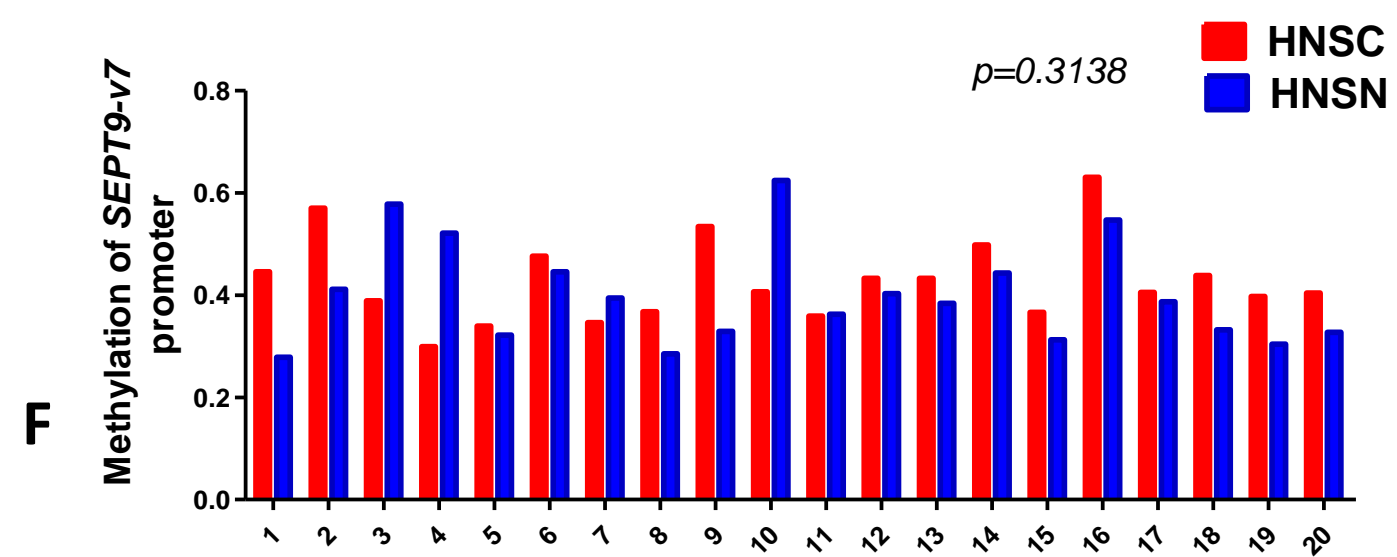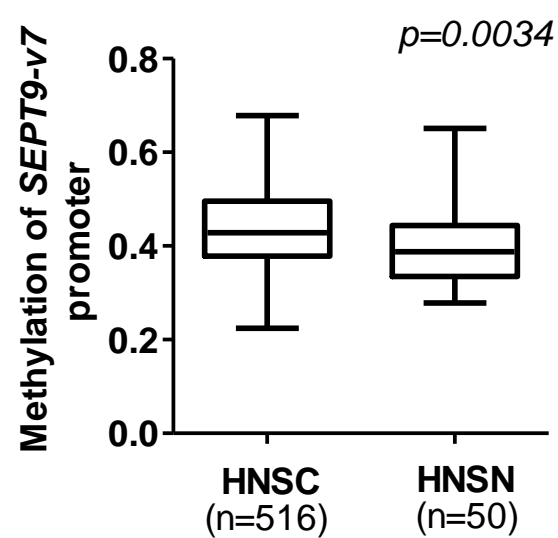

Supplement: Supplementary file 1 — Additional file 1: Figure S1. SEPT9_v1 and SEPT9_v3-7 promoter methylation in paired or independent HNSC and HNSN tissue samples or from MethHC database. (A) SEPT9_v1 promoter methylation in paired or independent HNSC and HNSN tissue samples. (B) SEPT9_v3 promoter methylation in paired or independent HNSC and HNSN tissue samples. (C) SEPT9_v4 promoter methylation in paired or independent HNSC and HNSN tissue samples. (D) SEPT9_v5 promoter methylation in paired or independent HNSC and HNSN tissue samples. (E) SEPT9_v6 promoter methylation in paired or independent HNSC and HNSN tissue samples. (F) SEPT9_v7 promoter methylation in paired or independent HNSC and HNSN tissue samples. [file 13148_2020_833_MOESM1_ESM.pdf]
